# Organisation and integrated healthcare approaches for people living with HIV, multimorbidity, or both: a systematic review

**DOI:** 10.1186/s12889-023-16485-y

**Published:** 2023-08-18

**Authors:** Vanessa Nicolau, Daniela Brandão, Tiago Rua, Ana Escoval

**Affiliations:** 1 NOVA National School of Public Health, Public Health Research Center, Comprehensive Health Research Center, CHRC, NOVA University Lisbon, Lisbon, Portugal; 2 King’s College London, London, UK

**Keywords:** Integrated care, People-centred care, HIV infection, Multimorbidity, Continuity of care, Self-management, Care coordination, Change management, Coproduction, Learning health systems

## Abstract

**Background:**

Universal recommendation for antiretroviral drugs and their effectiveness has put forward the challenge of assuring a chronic and continued care approach to PLHIV (People Living with HIV)*,* pressured by aging and multimorbidity. Integrated approaches are emerging which are more responsive to that reality. Studying those approaches, and their relation to the *what* of delivery arrangements and the *how* of implementation processes, may support future strategies to attain more effective organizational responses.

**Methods:**

We reviewed empirical studies on either HIV, multimorbidity, or both. The studies were published between 2011 and 2020, describing integrated approaches, their design, implementation, and evaluation strategy. Quantitative, qualitative, or mixed methods were included. Electronic databases reviewed cover PubMed, SCOPUS, and Web of Science. A narrative analysis was conducted on each study, and data extraction was accomplished according to the Effective Practice and Organisation of Care taxonomy of health systems interventions.

**Results:**

A total of 30 studies, reporting 22 different interventions, were analysed. In general, interventions were grounded and guided by models and frameworks, and focused on specific subpopulations, or priority groups at increased risk of poorer outcomes. Interventions mixed multiple integrated components. Delivery arrangements targeted more frequently clinical integration (*n* = 13), and care in proximity, community or online-telephone based (*n* = 15). Interventions reported investments in the role of users, through self-management support (*n* = 16), and in coordination, through multidisciplinary teams (*n* = 9) and continuity of care (*n* = 8). Implementation strategies targeted educational and training activities (*n* = 12), and less often, mechanisms of iterative improvement (*n* = 3). At the level of organizational design and governance, interventions mobilised users and communities through representation, at boards and committees, and through consultancy, along different phases of the design process (*n* = 11).

**Conclusion:**

The data advance important lessons and considerations to take steps forward from disease-focused care to integrated care at two critical levels: design and implementation. Multidisciplinary work, continuity of care, and meaningful engagement of users seem crucial to attain care that is comprehensive and more proximal, within or cross organizations, or sectors. Promising practices are advanced at the level of design, implementation, and evaluation, that set integration as a continued process of improvement and value professionals and users’ knowledge as assets along those phases.

**Trial registration:**

PROSPERO number CRD42020194117.

**Supplementary Information:**

The online version contains supplementary material available at 10.1186/s12889-023-16485-y.

## Background

The practised universal recommendation for antiretroviral drugs and the transformative power of their effectiveness has put forward interdependent challenges to current health systems, which are now more focused on monitoring side effects from the treatment and preventing age related health problems [1–10]. The growing demand and the increasing number of individuals on treatment, pressured by aging and multimorbidity, challenge the systems on how to assure a chronic and continued care approach to People Living with HIV (PLHIV) [1–10]*.* The management burden and system related barriers to access and navigation of services, increases the strain experienced by PLHIV, in addition to the issues of stigma and discrimination still prevalent in our societies [3, 4, 9, 10].

Acknowledging those challenges, the need for new models of care has been growing, and is now widespread at both national and international level [8, 11–13]*.* Health systems, backed by strong community-based resources, have pursued more integrated and people-centred care approaches. They incorporate principles of decentralization and proximity, coordination and flexibility, in order to provide services with more reach, which are adapted to national and local realities as well as evolving needs [1, 5, 14–18]*.* The main frameworks and models have been endorsed at a supranational level. They are supported by empirical evidence, and are referenced in strategic and health policy documents worldwide [13, 19–21]. There are several relevant frameworks and models, such as the chronic care model (CCM), the people-centred and integrated services and care, the shared care model, and the differentiated service delivery model (DSD). Each of these set a strategic structure of principles and mechanisms for responding to changing needs related to HIV and multimorbidity, but are no solution for all problems [16]*.* Integration and a people-centred organization of care and services should be looked at as dynamic and adaptive process. To maximize their effectiveness, interventions must employ robust implementation and change management strategies [3, 22]. Topics related to how these innovative approaches are put into practice have been emerging as a subject of research [3, 14, 18, 23]*.* Braithwaite et al. demand intersecting implementation science with complexity science as a means of putting attention on the dynamic properties of systems, and the need to manage multiple forces, variables and influences that interact within the process of change [23].

The International AIDS Society-Lancet Commission on the Future of Global Health and HIV Response, established in 2016, reinforce the real-world question of how [18]*.* An incremental step-based approach, as well as a learn by doing one, were proclaimed as a strategy to integrate HIV responses with the broader health system. Additionally, some determinants of success were highlighted. On the one hand, the need to preserve various features of the present HIV response, like the quality of care assured and respect for human rights, on the other hand, the need to include new features as participatory mechanisms for community inclusion and engagement in that change process [18]. That participatory element is linked, in the literature, to the identification of improvement opportunities and to the design of interventions, both resulting in access and acceptance gains [3, 8, 22]*.*

Future strategies to reshape organizational models and new responses can be supported by reviewing the available published literature on approaches to integrated services and people-centred care, which describe delivery arrangements in the fields of HIV, multimorbidity, or both, as well as how to manage change and measure their implementation process and effectiveness. Motivated by this, we conducted a systematic review to identify how international trends in organizational models are being incorporated into practice. This focused on how high-income countries respond to PLHIV, multimorbidity, or both, as well as their implementation and evaluation processes. This review is part of a larger project to rethink the contemporary model for responding to PLHIV in the Portuguese context.

## Methods

### Study design

We aimed to identify models and components of integrated healthcare approaches to PLHIV, multimorbidity, or both, and how these models are evaluated and applied in context. Our focus was not restricted to specific outcomes or to a level of integrated care (micro, meso or macro), rather it was more inclusive by being on models and components of integrated delivery arrangements targeting the complexity of care needs for PLHIV, multimorbidity or both, and the available evidence [24, 25]. Thus, a qualitative and descriptive strategy was adopted to extract data on two main domains: delivery arrangements and implementation strategies.

This systematic review was applied according to the updated preferred items for systematic reviews and meta-analyses (PRISMA 2020) statement [26, 27], which follows a pre-defined protocol approved by the International Prospective Register of Systematic Reviews (PROSPERO), whose identification number is CRD42020194117.

### Search strategy

Where applicable, the search strategy followed the PICOS framework (see Table 1). We searched MEDLINE (PubMed), SCOPUS, and Web of Science. The search strategy (see Additional file 1) was applied consistently, though with minor modifications for fulfilling the specific requirements of different databases. Boolean operators were used, linking the three main parts: HIV infection, multimorbidity, and care integration. To ensure the best capture of the empirical evidence related to experiences with integrated healthcare approaches, we conducted searches on a wide variety of terms, which from our previous experience, are used interchangeably. However, we did recognize the relevant conceptual differences between them. Additionally, we searched institutional sites of interest using a simplified search strategy: Joint United Nations Program on HIV/AIDS (UNAIDS) publications, British HIV Association—HIV Medicine Journal, NICE Evidence, European Observatory on Health Systems and Policies, Organisation for Economic Co-operation and Development (OECD) iLibrary, Health Programme Database – European Commission, Institutional Repository for Information Sharing (IRIS) – World Health Organization. Searches were also conducted on the references cited in selected papers in order to expand the literature base and assure a more comprehensive approach to the available evidence.

**Table 1:** Search strategy based on PICOS framework

**Population**	PLHIV or with multimorbidity (aged 18 + years)
**Intervention**	Interventions reporting healthcare approaches intended to integrate care at the micro (clinical), meso (organizational and/or professional) or macro (system) levels
**Comparator**	Not applicable
**Outcomes**	Three main outcomes: (1) Delivery arrangements defined by how, when, and where the care is provided, and who is delivering it; (2) Implementation strategies defined by the target of the intervention: organizational, human resources, area of practice, and settings; and (3) Identification of effect measures defined by indicators of the intervention’s impact, at the level of the process of care, quality of care and outcomes
**Study design**	No restrictions were applied to study designs. Studies published from January 1, 2011, onwards, using quantitative, qualitative, or mixed methods

Inclusion and exclusion criteria were defined and aligned according to the PICOS framework. The inclusion criteria applied: (a) studies published from January 1^st^, 2011, to July 22^nd^, 2020, (b) empirical studies describing the design, implementation, and/or evaluation of programmes or interventions intended to integrate care and services at the micro, meso or macro levels, (c) targeting adults living with HIV, multimorbidity, or both, (d) use quantitative, qualitative, or mixed methods approaches, and (e) studies from high-income countries (2020 world bank classification) and healthcare systems offering universal coverage or free HIV treatment. The exclusion criteria applied: (a) exclusively targeting individuals < 18 years, (b) theoretical studies focusing only on models or the concept of integrated care without reporting an actual intervention or practice, (c) studies focusing on a single disease approach for diseases other than HIV, (d) studies not written in English, (e) reports, conference abstracts, opinion pieces, editorials, dissertations, and research protocols, as well as (f) biomedicals studies.

### Data selection and extraction

Three researchers (AE, DB, VN) have taken part of the title and abstract screening stage. A complete dual independent review was conducted to identify the relevant articles against the inclusion and exclusion criteria. Eligibility criteria were pilot-tested, and no refinements appeared to be required. Where disagreements occurred, the third reviewer, was called in to resolve any issues if no consensus could be reached through discussion.

**Fig. 1:**
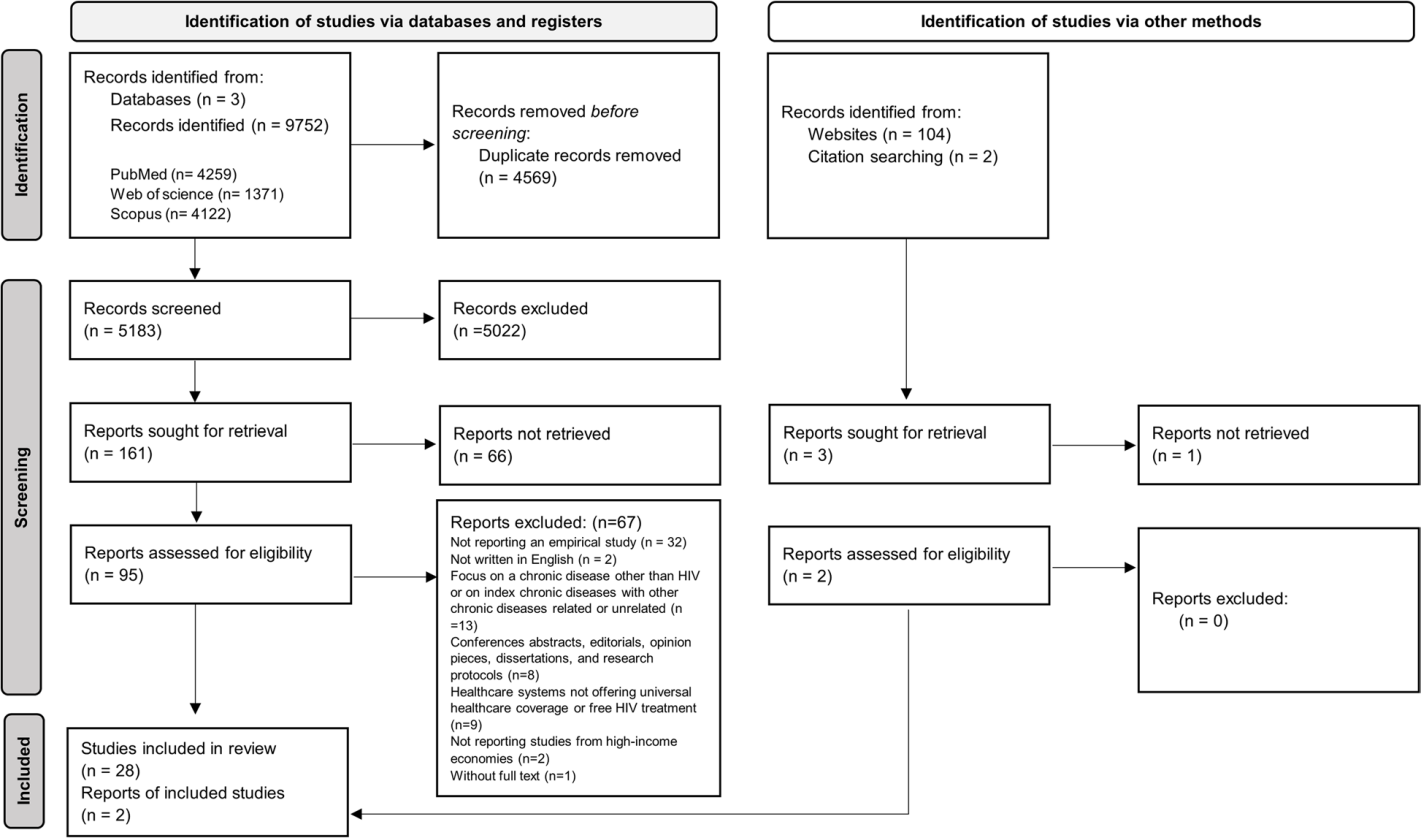
PRISMA flow diagram

A second phase of screening was executed independently by two researchers (DB, VN) based on the full-text analysis of the papers previously selected as relevant. We were able to reach a consensus for all divergences and differences of opinion. As such, it was not necessary to call in a third reviewer (AE) to act as arbiter as originally planned. The Rayyan software was used to upload the identified references and to manage the screening phases. This PRISMA flow diagram (see Fig. 1) summarizes the identification and screening phases [27].

Two researchers (DB, VN) extracted data using a standardized form developed for aggregating quality and content analysis (narrative synthesis). The forms were cross-checked by both researchers, and discrepancies were discussed till a consensus could be reached. The Effective Practice and Organisation of Care (EPOC) taxonomy of health systems interventions (2015) [28] was applied as a framework to extract data, focusing on two domains:

- (1) Delivery arrangements—changes in how, when and where healthcare is organised and delivered, and who delivers it. This covers four categories: (a) Where care is provided and any changes to the healthcare environment; (b) Who provides the care and how the healthcare workforce is managed; (c) Coordination of care and management of care processes; and (d) Information and communication technology.
- (2) Implementation strategies—Interventions designed to bring about changes in healthcare organizations, the behaviour of healthcare professionals or the use of health services by healthcare recipients. These cover three categories: (a) Interventions targeted at healthcare organisations; (b) Interventions that target healthcare workers; (c) Interventions targeted at specific types of practice, or conditions; and (d) user and community engagement.

Abstract information also included: the type of study, study design, context, referenced models/theoretical frameworks, primary and secondary objectives, level of integration, dimensions of lessons learned, and the type of measures extracted (see Additional files 2 and 3). Valentijn et al. classification was employed to categorize the level of integration: the macro level – system integration, the meso level – organizational and/or professional integration, and the micro level – clinical integration [25]. Type of measures followed the categorization: (a) process or intermediated outcomes of care – continuity, coordination, patient-centredness, management of lifestyle factors, management of specific diseases, medicine management, (b) use of health services, (c) experience of care and satisfaction, and (d) outcomes of care – patient-reported outcomes and adverse events [29]. An electronic spreadsheet (Microsoft Excel) supported the synthesis and analyse of extracted data.

### Quality assessment

Quality assessment was conducted via a three-step process, applied by two reviewers (DN, VN). The first step is choosing the appropriate categories of studies to appraise. The second is giving an independent score for each criterion, where “met” is classified as “yes”, and “not met” is classified as “no”, while “not enough information” is classified as “can´t tell”. Step three aims to resolve any disagreements via discussion. Procedures were supported by the validated Mixed Method Appraisal Tool ( MMAT) consisting of 19 core criteria in a quality scoring system grouped into five methodological categories [30–33]. The analysis focused on a detailed presentation of the ratings for each criterion classified as “no” or “can´t tell”, signalling a possible risk of bias, but no studies were excluded based on the degree of assessed quality.

## Results

### Characteristics of included studies

Our final sample included 30 studies, that underwent the descriptive analysis, corresponding to 22 independent integrated healthcare interventions (see Table 3). Of those, 12 studies on HIV, describing eight interventions, and 18 studies on multimorbidity, describing 14 interventions. Different studies covering the same intervention, offered different perspectives on analysis, from the implementation process to the evaluation of results, with different methodological approaches, and following the same or a different time interval. One study described an experience of multiple settings applying a common framework, across six European regions. It is called the CareWell Program [34].

Eligible interventions came from 13 different geographical locations. Spain (*n* = 6) and Canada (*n* = 4) had the highest number of initiatives (see Table 2). Integrated initiatives on HIV were reported in higher number from Australia (*n* = 2), and on multimorbidity from Spain (*n* = 5).

**Table 2:** Summary of geographical locations, publishing year and type of study, identified in the analysis

	**HIV**	**Multimorbidity**
**Geographical location**^**a**^		
Spain	1	5
England	0	3
Australia	2	1
Canada	1	3
Scotland	0	2
Croatia	0	1
Greece	1	0
Ireland	0	1
Italy	0	1
Netherlands	1	0
Norway	0	1
Poland	0	1
Taiwan	1	0
Wales	0	1
**Publishing year**^**b**^		
[2012–2015]	4	2
[2016–2019]	6	15
2020	1	2
**Type of study**^**b**^		
Quantitative approaches		
Q. Randomized controlled study	3	6
Q. Non-randomized study	5	6
Q. Descriptive study	1	1
Qualitative approaches	1	3
Mixed methods approaches	1	3

^a^per intervention

^b^per study

Different types of studies were identified, and the quantitative approaches prevailed in frequency (*n* = 22), over the qualitative (*n* = 4), and mixed methods approaches (*n* = 4) (see Table 2). Quantitative approaches focused on evaluating the effect of an intervention, compared to treatment as usual or with a pre and post-intervention design. Mixed methods approaches brought the perspectives of health care teams and users to the evaluation process. Qualitative approaches focused most on the processes of intervention design and change management, exploring the identification of factors that could hinder or promote more successful interventions, and of areas for optimization (see Additional file 2).

### Overview of integrated approaches

Overall, interventions were grounded and guided by models and frameworks, conceptual and/or empirical based (*n* = 18), exception made to four studies that do not make that information available (see Table 3).

**Table 3:** Summary of the characteristics considered per study

Study	Referenced models, theoretical framework	Targeted specific conditions	Level of integration	Delivery arrangements				Implementation strategies		Pilot tested
				**Setting**	**Who**	**Coordination and management of care process**	**Information and communication technology**	**At the healthcare workers**	**User and community engagement**	
[35]	x	over 18 years old, HIV infection, complex clinical management issues	meso—organizational /professional	tertiary referral HIV hospital		x			n.a	
[36]	x	over 18 years old, HIV infection, received primary care	meso—professional	urban community health centres	x	x	x	x	^b c^	
^a^ [37]	x	challenging and complex diagnoses, medically unexplained condition	meso—professional	part of Primary Health Care system	x	x			^b c^	x
^a^ [38]										
[39]	n.a	presence of three or more chronic diseases with complex degrees of severity	meso-organizational	integration of community-based and hospital services	x	x			n.a	
[40]	x	two or more chronic conditions, a minimum of four repeat medications, age over 18 years	micro—clinical	community-based	x			x	^b^	x
[41]	x	three or more chronic conditions, aged between 18 to 80 years	meso—professional	community-based PCPs unaffiliated with a primary care team		x	x		^b^	x
[42]	x	classified as multimorbid according to the stablished criteria, at least one hospitalization episode during the past year	meso—organizational /professional	primary healthcare centres together with the referral hospital	x	x			n.a	
^a^ [43]	x	aged between 30 and 65 years, two or more long-term conditions (type of condition not specified)	micro—clinical	primary care-based	x	x		x	^b c^	x
^a^ [44]										
^a^ [34]	n.a	aged 65 or older, a minimum of two chronic diseases (type of condition specified), classified as complex (severity, increased vulnerability, complex health needs, high risk of hospitalization, and/or intensive use of resources)	meso—organizational /professional	n.a	x	x	x		^b^	x
^a^ [45]	x	aged over 65, two or more chronic conditions, categorized as complex according to a risk stratification algorithm	meso—organizational/ professional	integrated care organizations	x	x	x		^b c^	
^a^ [46]	x	HIV-PWID (criteria for DSM-IV-TR substance dependence disorder), receiving simultaneous treatment for HIV and substance dependence, and psychosocial support	meso – professional	drug outpatient addiction centre		x			^b^	
^a^ [47]										
^a^ [48]	x	HIV-infected PWID – recent HIV infection and long-term HIV infection	micro—clinical	community based facilities	x	x		x	n.a	
^a^ [49]										
[50]	x	multimorbid frail, aged over 60 years, at risk for emergency (re) admissions	meso – professional/ organizational	cross-organizational (hospital and municipality)		x	x	x	^b c^	
[51]	x	aged 50 years or over, two or more long-term conditions	micro—clinical	community based facilities	x	x		x	^b^	
^a^ [52]	x	aged 18 years or older, at least three types of chronic condition (type of condition specified), grouped into ten types of condition with similar management considerations	meso—professional micro—clinical	general practices		x	x	x	^b c^	
^a^ [53]										
[54]	x	aged over 65, two or more self-reported long-term conditions, needing assistance with self-management (moderate activation)	micro—clinical	via telephone from a central NHS facility	x	x	x	x	^b c^	
[55]	x	aged 60 years or more, two or more chronic conditions, rated health differently from “very good” or “excellent”	micro—clinical	clinician led CDSMS program	x			x	^b^	
^a^ [56]	x	HIV, treatment experienced at risk of viral rebound or treatment-naive	micro – clinical	HIV clinics at academic and non-academic hospitals	x		x	x	^b^	x
^a^ [57]										
[58]	x	HIV, aged 30 or over, excluding those diagnosed with cardiovascular disease	micro—clinical	online	x	x	x		^b c^	
[59]	x	multiple diseases and/or on multiple medications, at the top of the risk stratification	micro—clinical	integrated organization—regional hospital and primary care centres	x	x			n.a	x
[60]	n.a	well controlled HIV patients (undetectable viral loads and follow-up coordinated between primary and secondary care)	micro—clinical	online	x		x		^b c^	x
[61]	x	multiple chronic conditions, social complexity	micro—clinical	primary care setting	x		x	x	^b c^	x
^a^ [62]	x	HIV diagnosis, male sex, age over 18 years	micro—clinical	online	x		x	x	^b c^	x
^a^ [63]										

*n. a*. not available

^a^ studies associated to the same intervention

^b^ engagement on direct care delivery; and

^c^ engagement on organizational design and governance

Approaches responding to the challenges and complexity of chronic diseases, such as the chronic care model (*n* = 6) [36–38, 50, 52, 53, 58, 59] the chronic disease self-management model (*n* = 4) [40, 55, 58, 61], and the Kaiser Permanente model (*n* = 1) were more frequently reported [42]. Along with those, holistic approaches, patient, client, or person-centred care models were identified as a main guidance for developing interventions (*n* = 6) [35, 37, 38, 41, 43, 44, 50–53]. Complementarily, some interventions applied mechanisms of change supported by theoretical approaches, such as behaviour change theories (*n* = 6) [40, 55–58, 62, 63]. Less often, frameworks and models guiding the complex change process were identified. They offered a structure that promote system-level thinking and participatory approaches. Two interventions on HIV used the Precede-proceed model (*n* = 2) [58, 62, 63]. Three interventions on multimorbidity reported using frameworks and models with that purpose, the UK Medical Research Council (MRC) complex intervention development framework (*n* = 2) [40, 43, 44] and the International Association for Public Participation (IAP2) framework (*n* = 1) [37, 38].

Interventions mainly described initiatives targeting the primary process of care delivery (micro level) (*n* = 13). These initiatives were anchored in the introduction of more holistic approaches, the expansion of narrower disease-focused guidelines and the reinforcement of the role of individuals as co-creators in the care process (see Table 3). Self-management programs, case management programs, and individual multidisciplinary care plans are some examples of these initiatives.

Eleven interventions were identified at the meso-level, anchored in building professional partnerships, within or across organisations (*n* = 9), and/or in building inter-organizational relationships (*n* = 6), either supported by hierarchical or network-like governance mechanisms.

Interventions combining different levels and dimensions of integration were identified. Combining the micro and meso level (professional) there were one intervention, and the dimensions of professional and organizational integration there were five interventions. Initiatives at the micro level were more frequent than other levels on multimorbidity interventions (*n* = 9), in the case of HIV interventions, initiatives at the micro and meso level were equally frequent (*n* = 4).

### Delivery arrangements and implementation strategies

Common to most studies, interventions described care in proximity through primary care, community care, online or telephone-based care (*n* = 15) (see Table 3). They invested in the role of users, their empowerment and capacity-building to self-manage health and well-being (*n* = 17); as well as in care coordination and the management process, by building and delivering care through multidisciplinary teams (*n* = 9) within or across organisations, and continuity of care (*n* = 8) (see Table 4). These investments had a point in common. That is, they were supported by enhanced communication between healthcare teams as well as between professionals and users. Also, these investments had a point of difference between HIV and multimorbidity interventions, which is expressed by the role assigned to primary care in endorsing solutions for proximity and continuity of care. HIV interventions were backed by strong community-based and hospital-based resources, rather than primary care-based resources.

**Table 4:** Overview of delivery arrangements and implementation strategies per HIV and multimorbidity interventions

	**Setting**	Hospital based	Cross organizations (hospital and primary care/municipality)	Primary care based	Community based	Online	Other			Total (N interventions)
**Delivery arrangements**	[HIV] [MM]	[2] []	[] [5]	[1] [7]	[2] [1]	[3] []	[] [1]			
	Total (N components)	2	5	**8**	3	3	1			
	**who provides care and how healthcare force is managed**	role expansion or task shifting	self-management	length of consultation						18
	[HIV] [MM]	[1] [2]	[7] [10]	[] [1]						[7] [11]
	Total (N components)	3	**17**	1						
	**coordination of care and management process**	disease management	integration	comprehensive geriatric assessment	discharge planning	continuity of care	shared decision-making	teams	case management	16
	[HIV] [MM]	[1] []	[1] []	[] [1]	[] [1]	[1] [7]	[] [3]	[3] [6]	[2] [3]	[5] [11]
	Total (N components)	1	1	1	1	**8**	3	**9**	5	
	**information and communication technology**	health information system	use of information and communication technology	telemedicine	smart home technologies					11
	[HIV] [MM]	[] []	[4] [4]	[] [1]	[1] [1]					[5] [6]
	Total (N components)	0	**8**	1	2					
**Implementation strategies**	**interventions at the healthcare workers**	local consensus processes	educational materials	monitoring the performance of the delivery of healthcare	educational outreach visits, or academic detailing	educational meetings	continuous quality improvement			11
	[HIV] [MM]	[1] [1]	[1] [3]	[] [1]	[] [2]	[4] [5]	[1] [1]			[4] [7]
	Total (N components)	2	4	1	2	**9**	2			
	**user and community engagement**	direct care delivery	organizational design and governance							17
	[HIV] [MM]	[6] [11]	[4] [7]							[6] [11]
	Total (N components)	**17**	11							

Delivery arrangements mixed multiple integrated components per intervention. The dimension of care coordination and management process totalized 29 components among 16 independent interventions, and the dimension of who provides care, 21 elements among 18 independent interventions (see Table 4).

Information and communication technologies were predominantly used to support delivery management of integrated care processes, through technology-based methods and tools to transfer and share healthcare information and support care (*n* = 8). Examples put into practice enablers that support key functions and activities for multidisciplinary collaboration, continuity of care, shared decision making and empowerment of PLHIV, or multimorbidity. These were realized via information and communication technology platforms, communication channels, standard electronic communication and discharge routines, electronic data entry templates, shared personal health folders, and computerized decision support systems.

Strategies directed to support effective change are commonly described as investments in education and training activities (*n* = 9), and less often, as mechanisms of iterative and continuous approaches to reviewing and improving transformation (*n* = 3). Ten of the 22 interventions described were pilot tested as a strategy to optimize and refine interventions, or as a trial for scaling up. Two of these were on HIV (see Table 3).

Generally, interventions focused on specific subpopulations, or priority groups at increased risk of poorer outcomes, rather than a population-based approach. A range of inclusion or exclusion criteria were applied. On HIV, three studies broadly approached people who were HIV infected, while the other nine interventions looked at specific subpopulations of PLHIV, such as persons who inject drugs, men who have sex with men, or other priority groups. This was accomplished via matched criteria, such as being HIV infected with complex clinical management issues (see Table 3). Variation between the criteria applied to the definition of multimorbidity was also identified. Interventions commonly applied the counting criteria with different cut-off points, either two or more, or three or more, combined chronic conditions. The criterion of age was used to restrict interventions to older populations, 60 or 65 years or older (*n* = 5), or from older populations (*n* = 2). Combined criteria were used to target individuals with more complex cases, needing a more intense allocation of resources and support in managing their health (*n* = 10), such as higher severity, vulnerability, higher risk of hospitalization, and social complexity.

### Collaborative processes—users and community engagement

Results profiled interventions supporting and promoting collaborative processes at the level of direct care delivery (*n* = 17) and the organizational design and governance level (*n* = 11) (see Table 4). At the level of direct care delivery three cross-cutting approaches were identified: 1) promoting and activating user skills for self-management (capacity building), 2) investing in tools and techniques for co-creation and shared decision-making with healthcare professionals (informing decisions), and 3) processes of trust-building within the relationship (strengthening relationships). At the level of organizational design and governance, users and community engagement followed two approaches, one where they performed as equal partners in planning and quality processes, by representation at boards, and committees, and the other where they performed as consultants, in needs assessment, validation processes, evaluation, and optimising interventions. The engagement arrangements designed were mainly based on samples of impacted people, where citizens more than stakeholders play an active role. These were on-site based and supported by deliberative processes.

HIV studies reported four independent interventions [36, 58, 60, 62, 63] combining direct care delivery, and organizational design and governance mechanisms, while for multimorbidity, there were seven [37, 38, 43–45, 50, 52–54, 61]. Solely targeting the level of direct care delivery, HIV reported two interventions [46, 47, 56, 57] and multimorbidity, four [40, 41, 51, 55].

### Measures and indicators reported

Generally, results-focused data were collected at the individual level, and measures and indicators reported cover different domains, from process to outcomes of care. At the level of the domains reported there were variations between HIV and multimorbidity studies (see Table 5). HIV studies focused on disease-specific measures related to disease management and medicines management, such as antiretroviral therapy uptake and viral load suppression. Multimorbidity studies focused on patient-centredness measures, such as patient activation, self-efficacy, and individualised goal attainment. Multimorbidity studies also focused on use of health services measures (*n* = 13), such as emergency room visits, unplanned admissions, length of stay due to unplanned admissions; and outcomes of care (*n* = 13), mainly patient-reported outcome measures (PROM), such as health-related quality of life and functional status. HIV studies also invested in measuring outcomes of care (*n* = 7), like HIV-related quality-of-life and mortality/survival rates.

**Table 5:** Measures and indicators reported per study

Type of indicators/measures	HIV	Multimorbidity	Total (N studies)
**Process of care**	**10**	11	**21**
Medicines management	7		
Management of specific diseases	7	3	
Management of lifestyle factors			
Patient centredness	2	11	
Comprehensiveness			
Coordination		2	
Continuity	3	2	
**Use of health services**	3	**13**	**16**
**Experience of care and satisfaction**	3	5	**8**
**Outcomes of care (PRO**^a ^**and adverse events)**	**7**	**13**	**20**

^a^ patient reported outcomes

## Discussion

This review summarized the empirical evidence published around approaches of integrated healthcare services for PLHIV, multimorbidity, or both. It focuses on the description of interventions, their implementation process and effectiveness measures.

Both points of difference and points of consensus were found in trends between interventions on HIV and on multimorbidity. Differences may represent divergences or varying rates of advance for integration. Two main differences were identified between HIV and multimorbidity interventions. For one, the role assigned to primary care in endorsing solutions for proximity and continuity of care. As to the other, the primacy attributed to measures and indicators monitoring and evaluating the care process, as well as the balance between disease specific and generic measures. Multimorbidity interventions assigned primacy to generic measures of the care process more in alignment with the vision of person-centred and integrated services and care. HIV studies focused on disease-specific measures related to disease management and medicines management.

Even though there are some points of difference in trends between HIV and multimorbidity interventions, consensus seem to prevail between strategies to attain integrated and people-centred services and care. From that, the data advance important considerations to take steps forward from disease or patient-focused care to integrated and people-centred care in response to the evolving needs and challenges of PLHIV throughout their life cycle. The analysis of the 30 studies that cover a total of 22 independent interventions support a discussion around three main themes: 1) delivery arrangements – levels and components implemented; 2) implementation processes – mechanisms and strategies implemented; and 3) coproduction – the role of users and communities, and mechanisms implemented.

### Delivery arrangements

Overall, results focus on the proximity and fluidity of care and services, bringing solutions closer to users, and targeting clinical integration, to overcome the paradigm of a disease-focused approach. When arrangements surpass the micro level or crosscut levels, interconnections and interdependencies reinforce the challenges associated to the change management process and the governance mechanisms putted into practice. The literature highlight complexity in relation to the challenges posed to professional and organizational integration, in terms of effectiveness and sustainability [24, 25]. Integration consistency should be built upon common ground: a shared paradigm of care and language, competencies, roles, responsibilities; and upon common governance, accountability and trust [24, 25, 64]. In support of that common ground, frameworks and models seem to play an important role, by guiding a shared philosophy of care, practice, and language, and structuring collaborative work for teams, including competencies, functions, and accountability.

Delivery arrangements evidenced a combination of multiple components per intervention, such as the building blocks for integration. Multidisciplinary collaboration, continuity of care and meaningful participation of users seem crucial for comprehensive and more proximal care, established within an organization, cross organizations, or cross sectors. That pattern seems to be in alignment with studies targeting the state of the art of people-centred and integrated services and care for PLHIV or multimorbidity, applying methodological approaches, which may be closer or more distant [7, 13, 65]. Some evidence related to context specificity and the organic emergence of local solutions highlight particular challenges connected to HIV, such as stigma, confidentiality concerns and minimum case load, that should be taken into account at the level of design, implementation and evaluation of integrated healthcare approaches [13, 20, 22].

Data converge with European results reporting higher investments in technology-based methods to transfer healthcare information and support the delivery of care [65]. The tools and elements in our data that were not reported on as often, such as smart home technologies [34] and telemedicine [41], were specially reinforced and tested in relation to the experience of the covid-19 pandemic [66–68]. This has the potential to enable access and proximity of services, as well as comprehensive care and empowerment of users.

### Implementation processes

Interventions reflect complexity, as a characteristic of the integrated healthcare approaches described, and as a feature of the context in which they are embedded. Acknowledging that assumption seems to impact the design, implementation, and research processes inherent to integrated healthcare approaches [23, 69–71].

Under that paradigm, Reed et al. set three main principles to guide practice and research: act scientifically and pragmatically, embrace complexity, and engage and empower [71]. Initiatives and mechanisms employed in the interventions under analysis crosscut those principles.

Near half of the approaches were grounded by a triangulation of conceptual basis, the available evidence, and the perspectives of professionals, the community and users, to attain a more context-specific problem-solving integrated approach [34, 36, 37, 43, 50, 52, 54, 56, 58, 60–62]. Strategies to adapt to system responses and generated learning were identified. Pilot testing was a common strategy linked to optimization and refinement of interventions [34, 38, 40, 41, 44, 56, 59–62]. Less commonly, continued improvement mechanisms [36, 50], and iterative monitoring delivery performance mechanisms were described [52]. Qualitative approaches were applied to deeply understand implementation processes [43, 53, 59, 61].

Multimorbidity approaches attributed primacy to the care process, through generic measures, more in alignment with the vision of person-centred and integrated services and care than HIV approaches. Both HIV and multimorbidity studies, invested in measure patient-reported outcomes, HIV-related, or generic, respectively. The perspective of users may contribute to decisions and actions acquainted with and linked to the vision of person-centred and integrated approaches, but may also work to empower users by making their preferences and needs heard [8, 72].

Overall, interventions recognised the agency power of the actors involved, from the side of supply to the side of demand. Meaningful engagement of users and professionals were reported [36, 43, 58, 59, 61], and support dedicated to opportunity and resources for that engagement were described [44, 45, 53, 61].

### Coproduction

Our empirical data on engagement of users and communities transverse a basis of partnership, shared decision-making, and commitment between all the participants, at the level of direct care delivery and at the level of organizational design and governance. Those activities were supported by tools, behavioural techniques, and participatory mechanisms.

The relation between the compromise of engagement and the value created by it is discussed in the literature [73–75]. Direct, instrumental value is recognized by organizations as efficiency, effectiveness, and innovation; and by citizens as satisfaction, need fulfilment, and empowerment. The identified experiences of user engagement echoed this association [37, 38, 40, 41, 44, 50, 58, 61]. The literature also recognises a societal value, through social learning, and by promoting a culture change at the organizational and citizenship levels [73].

That literature matches different levels of engagement intensity, information exchange practices and weight of influence to more discrete or structural outcomes in service redesign [74]. More discrete products, such as educational or tool development, informed policy or planning documents, are associated with consultative unidirectional feedback experiences. More structural outcomes, such as enhanced care processes or service delivery and governance, are associated with highly collaborative engagement experiences, such as co-design and partnership strategies [74]. Both mechanisms were identified in the analysis, either as consultative mechanisms [44, 45, 53, 58, 61–63] or as highly collaborative mechanisms [36–38, 50].

In the literature, that association is mediated by contextual factors that qualify user participation during the phases of design, delivery, and evaluation. These factors interact with strategies of engagement design and sampling [74, 75]. The set of the most common strategies associated with optimal engagement was identified. Making use of deliberative spaces, external facilitation, including users in all phases of the process, flexible approaches to engagement, user training, clarity of roles and objectives, offering feedback, leadership by local champions, institutional and executive-level commitment, dedicated resources from local authorities, and continued contact with management and executives [74]. Leadership seems key, but some authors signal a rise in clinician and community-led initiatives [74]. Time opportunity is also critical for meaningful engagement and should be considered when designing participatory mechanisms [74, 75].

### Practical lessons for designing, implementing and evaluating integrated care approaches

Results underlined two interdependent points for practical lessons, including methodological issues related to the scope of complex interventions research, their design, and evaluative strategy; as well as change management and implementation issues, related to the conceptualization of healthcare as a complex and adaptative system (see Additional file 3). Their elicitation recognizes that effectiveness can be impacted by intervention and by implementation deficiencies and failures.

Methodologically, the risks and biases highlighted can inform effective future integrated approaches. Results support the idea that interventions may not be suitable for all targeted individuals, and may not be effective in all contexts, highlighting the need to stratify interventions or adapt models to subpopulations and sub-groups specificities and consider sub-group analysis [35, 36, 42, 44, 47, 50–52, 54, 55]. Results also show that changes can be small and take a long time to show or reflect confounding effects, highlighting the need to determine the length of follow-up by weighing the rate and pattern of change, pondering the effect of the natural history of morbidity, and learning effects [39, 40, 45, 57, 62, 63]. Strategies need to consistently align the model of care with the underlying processes, the selected quality constructs, and their operationalization, resulting in measures that are robust in detecting and guiding change [36, 52, 60, 61]. Also, acknowledging interventions as a realm of variation in the number and combination of components and levels of integration, in the extent of behaviours targeted, in the required expertise and skills to perform accordingly, and in the number of practices and settings involved, adopting a whole practice approach in evaluation should be considered [45, 53].

Change management and implementation issues highlight the impact of the underlying organizational, social, political, and economic dimensions acting in the intervention context, and by that, strengthen the value of research dedicated to those processes [53, 58–61]. Investing in research for those processes produced evidence with regards to modifiable conditions linked to the success of interventions, impacting acceptancy, feasibility, scalability, and transferability across contexts [53, 61, 62]. Co-design strategies were implemented for developing and optimizing interventions in partnership with all key actors and stakeholders [37, 38, 43, 44, 58]. As enablers of a transformative environment at the practice level, they put education, training, and quality improvement tools into place [34]. Mechanisms to balance high fidelity to intervention design with adaptability to local conditions, were applied in some of the interventions under analysis, and should be putted into practice[34, 36, 53].

## Limitations of the study

The review covered a 10-year period, identifying and selecting studies published between January 2011 and July 2020. The timeframe was set to a realistic interval to capture trends and illustrate changes related to the assimilation of chronicity into the language of HIV, and translation into integrated and people-centred models of care.

Aware of the potential limitation from not searching in Embase associated to PubMed, due to its unavailability, a search strategy was defined in conjunction with a librarian to mitigate this limitation. The strategy includes the use of three databases, along with an enlarging the range of nomenclature considered (to reach a greater percentage of available citations and journals).

Even after employing that wide search strategy, the evidence for trends related to organizational models in high-income countries seem scarce. The topic cross boundaries for different disciplines and some alternative nomenclature may not have been considered or the lack of publishing in scientific journals may exert some influence [2, 76]. Also, the boundaries set by the inclusion and exclusion criteria may have contributed to the small number of eligible studies.

Even though the authors believe that results reflect the reality of the published integrated approaches targeting HIV and multimorbidity, which is in alignment with the objective for this review.

The level of disagreement between the reviewers was measured by the inter-rater agreement, with a value of 98,4%.

## Conclusion

Even with some points of difference in trends between HIV and multimorbidity interventions, the data advance important considerations to take steps forward from disease or patient-focused care, to integrated and people-centred care, in response to the evolving needs and challenges of PLHIV throughout their life cycle. Lessons learned focus initiatives at two critical levels: design and implementation.

Overall, results focus on the proximity and fluidity of care and services, bringing solutions closer to users and community facilities around clinical integration. Delivery arrangements mixed multiple elements per initiative, as building blocks for integration. Multidisciplinary collaboration, continuity of care and meaningful engagement of users seem crucial to attain comprehensive and more proximal care, within or across organizations, or sectors.

Interventions advance promising practices at the level of design, implementation, and evaluation, that set integration as a continued process of improvement and value professionals and users’ knowledge as assets along those phases.

Further research related to the conditions and strategies that work effectively for transformational change, sustained improvement, and scalability of people centred and integrated care is recommended in real-world conditions.

### Supplementary Information

**Additional file 1.** Search strategy detailing.

**Additional file 2.** Summary of abstracted data per study: geographical location and characteristics of studies.

**Additional file 3.** Summary of abstracted data per study: intervention design, implementation strategies, measures reported and lessons learned.

## Data Availability

The data generated or analysed that support the findings of this study are included in this published article and its supplementary information files. Any other data or materials are available from the corresponding author on reasonable request (VN: vnicolau@ensp.unl.pt).
