# Supplementary material for: Organisation and integrated healthcare approaches for people living with HIV, multimorbidity, or both: a systematic review
Source: BMC Public Health. 2023 Aug 18;23:1579. doi: 10.1186/s12889-023-16485-y (PMC10439547; doi:10.1186/s12889-023-16485-y)
Supplement: Supplementary file 1 — Additional file 1. Search strategy detailing. [file 12889_2023_16485_MOESM1_ESM.docx]

Additional file 1. Search strategy detailing.

(HIV Infections[MeSH] OR HIV[MeSH] OR hiv[tiab] OR hiv-1[tiab] OR hiv-2[tiab] OR hiv1[tiab] OR hiv2[tiab] OR “hiv infect*”[tiab] OR "human immunodeficiency virus"[tiab] OR "human immunedeficiency virus"[tiab] OR "human immuno-deficiency virus"[tiab] OR "human immune-deficiency virus"[tiab] OR “human immun*”[tiab]) OR (“Multimorb*”[tiab] OR “Multi-morb*”[tiab] OR “complex care needs”[tiab] OR "multiple chronic conditions"[tiab]) AND (“patient care manag*”[tiab] OR “Integrated Health Care System” [tiab] OR “Integrated Delivery System” [tiab] OR “Integrated care” [tiab] OR “integration of care” [tiab] OR “integrated care model*” [tiab] OR “disease management” [tiab] OR “chronic disease management” [tiab] OR “long term care” [tiab] OR “long-term care” [tiab] OR “chronic care model*” [tiab] OR “Organizational Innovation*” [tiab] OR “service innovation*” [tiab] OR “Clinical Pathway” [tiab] OR “care pathway” [tiab] OR “coordination of care” [tiab] OR “coordinated care” [tiab] OR “care coordination” [tiab] OR “multidisciplinary clinic” [tiab] OR “multidisciplinary center” [tiab] OR “Interdisciplinary Health Team*” [tiab] OR “Patient care team” [tiab] OR “Cascade of care” [tiab] OR “care cascade” [tiab] OR “treatment cascade” [tiab] OR “transitional care” [tiab] OR “Progressive Patient Care” [tiab] OR “Patient participation” [tiab] OR “Patient Involvement” [tiab] OR “Patient engagement” [tiab] OR “patient empowerment” [tiab] OR “patient activation” [tiab] OR “Self Management” [tiab] OR “self-management” [tiab] OR “Self-Care” [tiab] OR “self care” [tiab] OR “Integrated program*”[tiab] OR “differentiated service delivery” [tiab] OR “Patient-Focused Care” [tiab] OR “Patient Focused Care” [tiab] OR “people centered care” [tiab] OR “people-centered care” [tiab] OR “Patient-Centered Care” [tiab] OR “Patient Centered Care” [tiab] OR “Patient Navigation” [tiab] OR “Comprehensive Healthcare” [tiab] OR “Comprehensive Health care” [tiab] OR “Patient Care Continuity” [tiab] OR “Continuity of patient care” [tiab] OR “Continuum of Care” [tiab] OR “Care Continuum” [tiab] OR “Continuity of Care” [tiab] OR “Care Continuity” [tiab] OR “Continuous care” [tiab] OR “Patient Care Planning” [tiab] OR “Case Management” [tiab] OR “Managed care” [tiab] OR “Managed Care Program*”[tiab] OR “Collaborative care” [tiab] OR “collaborative care model” [tiab] OR “seamless care” [tiab] OR “shared care” [tiab]) AND ("2011"[PDAT]: "2020"[PDAT])​
