# Supplementary material for: Organisation and integrated healthcare approaches for people living with HIV, multimorbidity, or both: a systematic review
Source: BMC Public Health. 2023 Aug 18;23:1579. doi: 10.1186/s12889-023-16485-y (PMC10439547; doi:10.1186/s12889-023-16485-y)
Supplement: Supplementary file 2 — Additional file 2. Summary of abstracted data per study: geographical location and characteristics of studies. [file 12889_2023_16485_MOESM2_ESM.docx]

Additional file 2: Summary of abstracted data per study: geographical location and characteristics of studies.

| Year of publishing | Title | Geographical location | Type of study | Study design | Primary and secondary objectives | Study participants | Study quality assessment‡ |
| --- | --- | --- | --- | --- | --- | --- | --- |
|  |  |  |  |  |  |  |  |
| 2012 | Impact of the nurse-led case management program with retention in care on mortality among people with HIV-1 infection: a prospective cohort study. | Taiwan | Quantitative non-randomized study | Longitudinal prospective cohort study | Determine retention in care between 2005 and 2009, identify factors that contribute to retention in HIV care, and determine the impact of the CM model with retention in care on mortality among patients diagnosed with HIV/AIDS in Taiwan | Cohort of two groups, continuity of care (n = 261) vs. irregular/lost to follow-up (n = 314) | During the study period, is the intervention administered as intended (ct) |
|  |  |  |  |  |  |  |  |
| 2013 | Adoption of the chronic care model to improve HIV care: in a marginalized, largely aboriginal population. | Canada | Quantitative non-randomized study | Multisite, prospective, interventional cohort design | Measure the effectiveness of implementing the CCM to improve HIV clinical outcomes | 269 HIV-positive patients | The confounders are accounted for in the design and analysis (no) During the study period the intervention administered as intended (ct) |
| 2016 | †Meeting the needs of a complex population: a functional health- and patient-centered approach to managing multimorbidity | Canada | Quantitative descriptive study | Prevalence study and surveys | Describe the redesign strategies for care team planning, treatment approach, and patient inclusion | 456 individuals seen at the ICCS | the confounders accounted for in the design and analysis (ct) During the study period the intervention administered as intended (ct) |
| 2012 | †Proposed model of integrated care to improve health outcomes for individuals with multimorbidities |  | Quantitative non-randomized study | Experimental before and after study - pilot evaluation | Describe an integrated model of care to address the challenges of multimorbidity, and discuss the results of a pilot evaluation of this care model | Group of 20 patients | The confounders accounted for in the design and analysis (ct) During the study period the intervention administered as intended (ct) |
| 2017 | Case Management for Patients with Complex Multimorbidity: Development and Validation of a Coordinated Intervention between Primary and Hospital Care. | Spain | Quantitative non-randomized study | Retrospective cohort study using matched observations before and after the intervention | Analyse the effect of an integrated CM program designed for patients with complex multimorbidity in terms of healthcare resources utilisation. The hypothesis to be tested was whether improved continuity of care resulting from the integration of community-based and hospital services reduced the use of hospital resources amongst patients with complex multimorbidity. | Cohort of 714 adult patients admitted to the program | The confounders accounted for in the design and analysis (ct) During the study period, the intervention administered occurred as intended (ct) |
|  |  |  |  |  |  |  |  |
| 2015 | OPTIMAL, an occupational therapy led self-management support programme for people with multimorbidity in primary care: a randomized controlled trial. | Ireland | Quantitative randomized controlled trial | Pragmatic feasibility randomised controlled trial | Investigated the effectiveness of an occupational therapy led self-management support programme, OPTIMAL for increasing activity participation in individuals with multimorbidity. | 63 individuals referred to the programme | Outcome assessors not blinded to the intervention provided (-) |
| 2019 | Connecting People With Multimorbidity to Interprofessional Teams Using Telemedicine. | Canada | Mixed method study | Case study and quantitative analysis of TIP uptake, satisfaction survey, and a qualitative thematic analysis of participant feedback | Evaluate the feasibility of the novel approach performing a case study of TIP—Telemedicine IMPACT Plus—a 1-time interprofessional consultation with primary care physicians (PCPs) and their patients. And assess the satisfaction with the TIP intervention | 76 patients referred from 53 PCPs and 4 emergency departments, and 65 PCPs participated in TIP |  |
|  |  |  |  |  |  |  |  |
|  |  |  |  |  |  |  |  |
| 2016 | Cost-utility analysis of an integrated care model for multimorbid patients based on a clinical trial. | Spain | Quantitative randomized controlled trial | Cost-utility analysis based on a prospective and multi-centre cluster randomized trial, with two groups randomized by doctor’s list | Cost-utility analysis on an integrated healthcare model for patients with multimorbidity, compared to a conventional reactive healthcare system - based on episodic reactive care. | 140 patients recruited | Outcome assessors are blinded to the intervention provided (ct) |
|  |  |  |  |  |  |  |  |
| 2016 | †The CARE Plus study - a whole-system intervention to improve quality of life of primary care patients with multimorbidity in areas of high socioeconomic deprivation: exploratory cluster randomised controlled trial and cost-utility analysis. | Scotland | Quantitative randomized controlled trial | Phase 2 exploratory cluster randomised controlled trial and a cost-utility analysis | Evaluate a whole-system primary care-based complex intervention. Test the feasibility and likely cost-effectiveness of the intervention in preparation for a large-scale definitive RCT. | 225 eligible patients, 152 (68 %) participated and 67/76 (88 %) in each arm completed the 12-month assessment |  |
|  |  |  |  |  |  |  |  |
|  |  |  |  |  |  |  |  |
|  |  |  |  |  |  |  |  |
| 2016 | †The development and optimisation of a primary care-based whole system complex intervention (CARE Plus) for patients with multimorbidity living in areas of high socioeconomic deprivation |  | Qualitative study | Qualitative description - six group discussions and a pilot evaluation | Describe the further development and co-design of the intervention and its optimisation following piloting in two practices located in the high deprivation areas | 30 suitable patients identified for the pilot of the CARE Plus intervention, and 20 patients agreed to participate (14 from practice A and six from practice B) |  |
|  |  |  |  |  |  |  |  |
| 2020 | †Impact assessment of an innovative integrated care model for older complex patients with multimorbidity: The carewell project | 6 European regions: Basque Country (Spain), Zagreb (Croatia), Lower Silesia (Poland), Veneto (Italy), Puglia (Italy), and Powys (UK - Wales). | Quantitative non-randomized study | Quasi-experimental study, multicentric, using intervention and control groups | Evaluate the impact in terms of use of health services, clinical outcomes, functional status, and patient’s satisfaction of an integrated care program, the CareWell program, for complex patients with multimorbidity in six European regions. | 856 patients recruited (475 intervention and 381 control) | During the study period, is the intervention administered (or exposure occurred) as intended (ct) |
| 2020 | †Impact of the CareWell integrated care model for older patients with multimorbidity: a quasi-experimental controlled study in the Basque Country | Spain | Mix- methods study | Pilot study - quasi experimental study using intervention and control groups - semi-structured interviews to assess satisfaction and mixed regression models to measure the effect of the intervention | Evaluate the impact of CareWell integrated care model for older patients with multimorbidity in the Basque Country, in terms of use of health resources, clinical effectiveness, patient functional and mental status and satisfaction. | 200 patients recruited (101 intervention and 99 control) |  |
|  |  |  |  |  |  |  |  |
| 2015 | †Low Non-structured Antiretroviral Therapy Interruptions in HIV-Infected Persons Who Inject Drugs Receiving Multidisciplinary Comprehensive HIV Care at an Outpatient Drug Abuse Treatment Center | Spain | Quantitative non-randomized study | Clinical cohort - longitudinal study | Evaluate the incidence of patient-initiated non-structured treatment interruptions in HIV-infected persons who inject drugs and who received a multidisciplinary comprehensive program, including medical HIV care, drug-dependence treatment, and psychosocial support, at a drug outpatient addiction centre | 132 patients included |  |
| 2020 | †Reaching the 909090 UNAIDS treatment target for people who inject drugs receiving integrated clinical care at a drug-use outpatient treatment facility |  | Quantitative descriptive study | Descriptive cross-sectional study | Describe the effectiveness of an integrated care model to reach the UNAIDS 90–90–90 target | 84 patients included | Is the sample representative of the target population? (ct) |
| 2019 | †Identifying, linking, and treating people who inject drugs and were recently infected with HIV in the context of a network-based intervention | Greece | Quantitative non-randomized study | Before and after study – interviews followed a structured questionnaire | Describe and depict the continuum of care of PWID diagnosed with recent HIV infection in Athens, Greece in the context of a risk network-based project with a comprehensive case management component | 45 recently HIV-infected PWID (23 seeds and 22 network members of seeds) | Are the confounders accounted for in the design and analysis (no) |
|  |  |  |  |  |  |  |  |
| 2016 | †A network intervention that locates and intervenes with recently HIV-infected persons: The Transmission Reduction Intervention Project (TRIP) |  | Quantitative non-randomized study | Cohort study | Evaluate a network intervention to detect individuals recently infected | 23 RS, 171 network members of the RS, 19 LCS, and 65 network members of the LCS |  |
| 2019 | Person-centred, integrated and pro-active care for multi-morbid elderly with advanced care needs: A propensity score-matched controlled trial | Norway | Quantitative non-randomized study | Synthetic RCT - a pragmatic prospective, controlled clinical trial based on hospital electronic health record data | Analyse the effect of a synergistic intervention, which implements: 1) Person-centred goal-oriented care, with 2) Integrated Care and 3) pro-active care. | based on hospital electronic health record data of 439 individuals and 779 propensity score matched controls |  |
| 2019 | Does a social prescribing 'holistic' link-worker for older people with complex, multimorbidity improve well-being and frailty and reduce health and social care use and costs? A 12-month before-and-after evaluation | UK (England) | Quantitative non-randomized study | Before-and-after study - followed people receiving the intervention | Evaluate the impact of ‘holistic’ link-workers on service users’ well-being, activation and frailty, and their use of health and social care services and the associated costs. And explore what patient characteristics on programme entry were associated with positive outcomes | 126 participants with health outcomes data. Only 86 (68%) with a full 12 months’ worth of health and social care data |  |
|  |  |  |  |  |  |  |  |
| 2018 | †Management of multimorbidity using a patient-centred care model: a pragmatic cluster-randomised trial of the 3D approach | UK (England and Scotland) | Quantitative randomized controlled trial | Pragmatic cluster-randomised trial conducted | Implement, at scale, a new approach (3D) to managing patients with multimorbidity in primary care and to assess its effectiveness | 1546 patients recruited, assigned to intervention (n=797) or usual care (n=749) |  |
| 2019 | †Can implementation failure or intervention failure explain the result of the 3D multimorbidity trial in general practice: Mixed-methods process evaluation |  | Mix method study | Cross-trial data. Interviews, focus groups and review observations data were integrated with quantitative data about implementation. interviews were applied before and after trial | Examine whether the measured lack of effect on the primary outcome in the 3D trial was due to implementation and/or intervention failure | 11 general practitioners, 14 nurses, 7 administrators and 38 patients from 9 of 16 intervention practices | Are divergences and inconsistencies between quantitative and qualitative results adequately addressed (ct) |
| 2018 | Is telephone health coaching a useful population health strategy for supporting older people with multimorbidity? An evaluation of reach, effectiveness and cost-effectiveness using a 'trial within a cohort' | UK (England) | Quantitative randomized controlled trial | Pragmatic, two-arm, patient-level, randomised trial. All outcomes were collected at four time points across the study: at baseline, then at 6, 12 and 20 months | Assess the cost-effectiveness of health coaching for patients with multimorbidity | 504 older individuals selected for health coaching, and 41% consented. More than 80% of consenters received the defined ‘dose’ of 4+ sessions | Are outcome assessors blinded to the intervention provided? (no) Did the participants adhere to the assigned intervention? (no) |
|  |  |  |  |  |  |  |  |
|  |  |  |  |  |  |  |  |
|  |  |  |  |  |  |  |  |
| 2018 | A self-management support program for older Australians with multiple chronic conditions: A randomised controlled trial | Australia (South Australia) | Quantitative randomized controlled trial | Randomised controlled trial comparing the effectiveness of the intervention with outcomes of an attention-based control program. Outcome measures were administered pre and post-intervention | Determine whether a clinician-led chronic disease self-management support (CDSMS) program improves the overall self-rated health level of older Australians with multiple chronic health conditions. And assess its perceived usefulness in improving their management of health, and whether the program had affected the relationships of participants with their GPs | 254 participants of whom 231 (117 control and 114 CDSMS participants) completed the 6-month programs and provided complete outcomes data (91%) |  |
|  |  |  |  |  |  |  |  |
|  |  |  |  |  |  |  |  |
| 2017 | †Effectiveness and cost-effectiveness of a nurse-delivered intervention to improve adherence to treatment for HIV: a pragmatic, multicentre, open-label, randomised clinical trial | Netherlands | Quantitative randomized control trial | Pragmatic, multicentre, open label, randomised controlled trial. Randomly assigned participants (1:1) to either intervention or treatment as usual. Outcomes collected at three timepoints (months 5, 10, and 15) | Examined the effectiveness and cost-effectiveness of the Adherence Improving self-Management Strategy (AIMS) compared with treatment as usual | 221 patients identified (intent to treat sample); 109 assigned to AIMS and 112 to treatment as usual |  |
|  |  |  |  |  |  |  |  |
| 2019 | †Cost-effectiveness and Cost-utility of the Adherence Improving Self-management Strategy in Human Immunodeficiency Virus Care: A Trial-based Economic Evaluation. |  | Quantitative randomized control trial | Trial-based economic evaluation, piggy-backed on the multicentre trial, from a societal perspective, over the 15-month trial follow-up period | Examine the cost -effectiveness and cost-utility of the AIMS compared to treatment as usual (TAU) |  | Are outcome assessors blinded to the intervention provided? (ct) Did the participants adhere to the assigned intervention? (no) |
|  |  |  |  |  |  |  |  |
| 2018 | The systematic development of a complex intervention: HealthMap, an online self-management support program for people with HIV | Australia | Qualitative study | Descriptive study of the systematic approach to the development of the intervention and its outcomes via concept mapping technique, workshops, online surveys, interviews, and literature reviews | Outline the systematic process used to design and develop the HealthMap program, prior to its evaluation in a cluster-randomised trial | n.a. |  |
|  |  |  |  |  |  |  |  |
|  |  |  |  |  |  |  |  |
|  |  |  |  |  |  |  |  |
| 2016 | Qualitative Evaluation of the Implementation of an Integrated Care Delivery Model for Chronic Patients with Multi-Morbidity in the Basque Country | Spain (Basque country) | Qualitative study | Nominal group technique applied to four groups of professionals. | Evaluate, from the participants’ point of view, the model of integration in the Goierri-Alto Urola Integrated Healthcare Organisation and propose areas of improvement | n.a. |  |
|  |  |  |  |  |  |  |  |
| 2016 | Feasibility and Preliminary Outcomes of a Web and Smartphone-Based Medication Self-Management Platform for Chronically Ill Patients. | Spain (Barcelona) | Quantitative non-randomized study | Single-arm prospective pre-post intervention study (pilot study) | Assess the feasibility and preliminary outcomes of a medication self-management platform for chronically ill patients, Medplan | 42 patients completed the study. Eight HIV-infected patients | Are the participants representative of the target population? (ct) Are the confounders accounted for in the design and analysis? (no) |
|  |  |  |  |  |  |  |  |
| 2018 | mHealth Tools for the Self-Management of Patients With Multimorbidity in Primary Care Settings: Pilot Study to Explore User Experience | Canada (Toronto) | Qualitative study | Secondary analysis of a 4-week pilot study. Focus groups post-intervention and thematic analyses | Explore the experience and expectations of patients with multimorbidity and their providers around the use of the ePRO tool in supporting self-management efforts | n.a. |  |
| 2015 | †Online self-management for gay men living with HIV: a pilot study | Australia | Mix method study | Single-arm pilot feasibility study followed by a pilot randomised controlled trial. Pilot 1 employed a pre and post-test design. Pilot 2 employed a within- and between-group differences. Primary outcomes were evaluated at three time-points (baseline, post-intervention and 12-week’s post-intervention follow-up). Qualitative assessment - Interviews at completion of the program | Assess the feasibility, acceptability and effectiveness of Positive Outlook, compared to a ‘usual care’ control | Pilot 1. ten participants included. Data collected from nine participants. Six also interviewed about their experience. Pilot 2. 37 participants included, allocated to intervention group (n = 17) and control group (n = 18). Two participants withdrew. Data collected from 21 participants post intervention. Four participants interviewed and seven completed the feedback questionnaire |  |
|  |  |  |  |  |  |  |  |
|  |  |  |  |  |  |  |  |
|  |  |  |  |  |  |  |  |
| 2016 | †The Positive Outlook Study: A Randomised Controlled Trial Evaluating Online Self-Management for HIV Positive Gay Men |  | Quantitative randomised controlled trial | Randomised controlled trial. Primary outcome evaluated at three time-points (baseline, post-intervention, and 12-week’s post-intervention follow-up) | Evaluate the effectiveness of an online self- management program in improving health outcomes and well-being for gay men living with HIV in Australia | 132 participants allocated to the intervention (n = 68) or usual care control (n = 64) groups |  |
|  |  |  |  |  |  |  |  |
|  |  |  |  |  |  |  |  |

Legend of table: † studies associated to the same intervention; n.ap. not applicable; (ct) can´t tell; ‡ only no and can´t tell rates are presented.
