# Supplementary material for: Organisation and integrated healthcare approaches for people living with HIV, multimorbidity, or both: a systematic review
Source: BMC Public Health. 2023 Aug 18;23:1579. doi: 10.1186/s12889-023-16485-y (PMC10439547; doi:10.1186/s12889-023-16485-y)
Supplement: Supplementary file 3 — Additional file 3. Summary of abstracted data per study: intervention design, implementation strategies, measures reported and lessons learned. [file 12889_2023_16485_MOESM3_ESM.docx]

Additional file 3: Summary of abstracted data per study: intervention design, implementation strategies, measures reported and lessons learned.

| **Title** | **Referenced models, theoretical framework** | **Targeted specific conditions** | **Level of integration** | **Delivery arrangements** | | | | **Implementation strategies** | | **Type of measures/indicators** | **Dimensions of lessons learned** |  |
| --- | --- | --- | --- | --- | --- | --- | --- | --- | --- | --- | --- | --- |
|  |  |  |  | Where care is provided | Who provides care and how the healthcare workforce is managed | Coordination of care and management of care process | Information and communication technology | interventions at the healthcare workers | user and community engagement |  |  | **Pilot tested** |
| Impact of the nurse-led case management program with retention in care on mortality among people with HIV-1 infection: a prospective cohort study | Client-centred model | over 18 years old with HIV infection, and complex clinical management issues |  | tertiary referral HIV hospital |  | 1. case management program |  |  | n.a. | Process of care | Design of interventions |  |
|  |  |  | Meso - organizational and professional |  |  |  |  |  |  | Outcomes of care | Selection of beneficiaries and allocation of intervention to practices |  |
|  |  |  |  |  |  |  |  |  |  |  | Size and timing of effects |  |
|  |  |  |  |  |  |  |  |  |  |  |  |  |
| Adoption of the chronic care model to improve HIV care: in a marginalized, largely aboriginal population | Chronic care model | over 18 years old with HIV infection who received primary care | Meso - professional | urban community health centres | 1. Role expansion or task shifting  2. Self-management | 1. Disease management 2. Teams | 1. The use of information and communication technology | 1. Continuous quality improvement 2. Educational meetings 3. Local consensus processes | 1. direct care delivery 2. organizational design and governance - PLHIV community advisory committee | Process of care | Design of interventions |  |
|  |  |  |  |  |  |  |  |  |  | Outcomes of care | Selection of beneficiaries and allocation of intervention to practices |  |
|  |  |  |  |  |  |  |  |  |  |  | Operationalization of measures |  |
|  |  |  |  |  |  |  |  |  |  |  | Size and timing of effects |  |
|  |  |  |  |  |  |  |  |  |  |  |  |  |
| †Meeting the needs of a complex population: a functional health- and patient-centered approach to managing multimorbidity | Chronic Care Model | challenging and complex diagnoses and a medically unexplained condition | Meso - professional | integral part of Primary Health Care system | 1. self-management | 1. case management  2. shared decision-making 3. Teams |  |  | 1. direct care delivery  2. organizational design and governance - equal partners and members in Quality Teams | Process of care | Operationalization of measures | x |
|  | International Classification of Functioning, Disability and Health (WHO) |  |  |  |  |  |  |  |  | Use of health services | Change management and implementation -acceptancy and feasibility |  |
|  | Whole-person model of care for complex populations |  |  |  |  |  |  |  |  | Outcomes of care |  |  |
|  | Primary Health Care Competency Framework |  |  |  |  |  |  |  |  |  |  |  |
|  | Canadian Competency Framework for Interprofessional Collaboration |  |  |  |  |  |  |  |  |  |  |  |
| †Proposed model of integrated care to improve health outcomes for individuals with multimorbidities | The IAP2 Framework - International Association for Public Participation |  |  |  |  |  |  |  |  | Process of care Experience of care and satisfaction | Design of interventions |  |
|  |  |  |  |  |  |  |  |  |  | Outcomes of care | Selection of beneficiaries and allocation of interventions to practices |  |
|  |  |  |  |  |  |  |  |  |  |  | Size and timing of effects |  |
|  |  |  |  |  |  |  |  |  |  |  | Change management and implementation – acceptancy and feasibility |  |
|  |  |  |  |  |  |  |  |  |  |  |  |  |
| Case Management for Patients with Complex Multimorbidity: Development and Validation of a Coordinated Intervention between Primary and Hospital Care | n.a. | presence of three or more chronic diseases with complex degrees of severity | Meso - organizational | integration of community-based and hospital services | 1. self-management | 1. case management 2. Teams |  |  | n.a. | Use of health services | Design of interventions |  |
|  |  |  |  |  |  |  |  |  |  | Outcomes of care | Selection of beneficiaries and allocation of interventions to practices |  |
|  |  |  |  |  |  |  |  |  |  |  | Size and timing of effects |  |
|  |  |  |  |  |  |  |  |  |  |  |  |  |
| OPTIMAL, an occupational therapy led self-management support programme for people with multimorbidity in primary care: a randomized controlled trial | UK Medical Research Council Framework | two or more chronic conditions and a minimum of | Micro - clinical | Community-based | 1. self-management |  |  | 1. educational materials  2. educational meetings | 1. direct care delivery | Process of care Use of healthcare services | Selection of beneficiaries and allocation to intervention | x |
|  | Stanford Chronic Disease Self-Management  Programme | four repeat medications, and age over 18 years |  |  |  |  |  |  |  | Experience of care and satisfaction | Operationalization of measures |  |
|  | Behavioural change theories |  |  |  |  |  |  |  |  | Outcomes of care | Size and timing of effects |  |
|  |  |  |  |  |  |  |  |  |  |  | Change management and implementation – feasibility, scalability, and transferability |  |
|  |  |  |  |  |  |  |  |  |  |  |  |  |
| Connecting People with Multimorbidity to Interprofessional Teams Using Telemedicine | Canadian National Competency Framework | three or more chronic conditions, aged between 18 to 80 years | Meso - professional | community-based PCPs unaffiliated with a primary care team |  | 1. shared decision making  2. teams | 1. telemedicine |  | 1. direct care delivery | Process of care Use of health services | Design of interventions | x |
|  | Patient-centred approach |  |  |  |  |  |  |  |  | Experience of care and satisfaction | Operationalization of measures |  |
|  |  |  |  |  |  |  |  |  |  |  | Change management and implementation issues – feasibility, scalability, and transferability |  |
|  |  |  |  |  |  |  |  |  |  |  |  |  |
| Cost-utility analysis of an integrated care model for multimorbid patients based on a clinical trial | Kaiser Permanente model | classified as multimorbid according to the stablished criteria, who have at least one hospitalization episode during the past year | Meso l- organizational and professional | primary healthcare centres together with the referral hospital | 1. self-management | 1. continuity of care |  |  | n.a. | Use of health services | Design of interventions |  |
|  |  |  |  |  |  |  |  |  |  | Outcomes of care | Selection of beneficiaries and allocation of intervention to practices |  |
|  |  |  |  |  |  |  |  |  |  |  |  |  |
| †The CARE Plus study - a whole-system intervention to improve quality of life of primary care patients with multimorbidity in areas of high socioeconomic deprivation: exploratory cluster randomised controlled trial and cost-utility analysis | UK Medical Research Council framework | aged between 30 and 65 years and with two or more long-term conditions (type of condition not specified) | Micro - clinical | primary care-based | 1. Length of consultation 2. self-management |  |  | 1. educational meetings  2. educational materials | 1. direct care delivery 2. organizational design and governance - develop and optimize interventions - co-design discussion groups and interviews | Process of care Use of health services | Selection of beneficiaries and allocation of intervention to practice | x |
|  | 5 A’s approach |  |  |  |  |  |  |  |  | Outcomes of care | Operationalization of measures |  |
|  | CARE approach |  |  |  |  |  |  |  |  |  | Size and timing of effects |  |
|  |  |  |  |  |  |  |  |  |  |  | Change management and implementation – acceptancy and feasibility |  |
| †The development and optimisation of a primary care-based whole system complex intervention (CARE Plus) for patients with multimorbidity living in areas of high socioeconomic deprivation |  |  |  |  |  | 1. continuity of care |  |  |  | n.ap. | Size and timing of effects |  |
|  |  |  |  |  |  |  |  |  |  |  | Change management and implementation – acceptancy and feasibility |  |
| †Impact assessment of an innovative integrated care model for older complex patients with multimorbidity: The carewell project | n.a. | aged 65 or older, a minimum of two chronic diseases (type of condition specified), classified as complex, considering (severity, increased vulnerability, complex health needs, high risk of hospitalization, and/or intensive use of resources) | Meso - | n.a. | 1. Role expansion or task shifting 2. self-management | 1. Continuity of care 2. Teams | 1. health information system 2. The use of information and communication technology 3. smart health technologies  4. telemedicine |  | 1. direct care delivery | Process of care Use of health services | Design of interventions | x |
|  |  |  | organizational and professional |  |  |  |  |  |  | Outcomes of care | Size and timing of effects |  |
|  |  |  |  |  |  |  |  |  |  |  | Change management and implementation – acceptancy, feasibility, and transferability |  |
| †Impact of the CareWell integrated care model for older patients with multimorbidity: a quasi-experimental controlled study in the Basque Country | CareWell integrated care model | aged over 65 with two or more chronic conditions, categorized as complex according to a risk stratification algorithm | Meso - | integrated care organizations | 1. Role expansion or task shifting 2. self-management | 1. Continuity of care 2. Teams | 1. use of information and communication technology |  | 1. involved in direct care delivery 2. organizational design and governance | Process of care | Design of interventions |  |
|  |  |  | organizational and professional |  |  |  |  |  |  | Use of health services | Selection of beneficiaries and allocation of intervention to practices |  |
|  |  |  |  |  |  |  |  |  |  | Experience of care and satisfaction | Size and timing of effects |  |
|  |  |  |  |  |  |  |  |  |  | Outcomes of care |  |  |
| †Low Non-structured Antiretroviral Therapy Interruptions in HIV-Infected Persons Who Inject Drugs Receiving Multidisciplinary Comprehensive HIV Care at an Outpatient Drug Abuse Treatment Center | UNAIDS Global AIDS Monitoring Guidelines | HIV-PWID (criteria for DSM-IV-TR substance dependence disorder) receiving simultaneous treatment for HIV and substance dependence, and psychosocial support | Meso – professional | drug outpatient addiction centre |  | 1. Teams 2. Integration |  |  |  | Process of care | Design of interventions |  |
|  |  |  |  |  |  |  |  |  | 1. direct care delivery |  | Selection of beneficiaries and allocation of intervention to practices |  |
| †Reaching the 909090 UNAIDS treatment target for people who inject drugs receiving integrated clinical care at a drug-use outpatient treatment facility |  |  |  |  |  |  |  |  |  | Process of care | Design of interventions |  |
|  |  |  |  |  |  |  |  |  |  |  | Selection of beneficiaries and allocation of intervention to practices |  |
|  |  |  |  |  |  |  |  |  |  |  | Operationalization of measures |  |
|  |  |  |  |  |  |  |  |  |  |  | Size and timing of effects |  |
|  |  |  |  |  |  |  |  |  |  |  |  |  |
| †Identifying, linking, and treating people who inject drugs and were recently infected with HIV in the context of a network-based intervention | Behavioural change techniques | HIV-infected PWID – recent HIV infection and long-term HIV infection | Micro - clinical | community based facilities | 1. self-management | 1. case management  2. teams |  | 1. educational meetings | n.a. | Process of care | Design of interventions |  |
|  | Centre for Disease Control and Prevention guidelines |  |  |  |  |  |  |  |  |  | Selection of beneficiaries and allocation of intervention to practices |  |
|  |  |  |  |  |  |  |  |  |  |  | Operationalization of measures |  |
| †A network intervention that locates and intervenes with recently HIV-infected persons: The Transmission Reduction Intervention Project (TRIP) |  |  |  |  |  |  |  |  |  | Process of care | Design of interventions |  |
|  |  |  |  |  |  |  |  |  |  |  | Selection of beneficiaries and allocation to intervention practices |  |
|  |  |  |  |  |  |  |  |  |  |  |  |  |
| Person-centred, integrated and pro-active care for multi-morbid elderly with advanced care needs: A propensity score-matched controlled trial | Chronic Care Model | multimorbid frail aged over 60 years at risk for emergency (re) admissions | Meso - professional and organizational | cross-organizational (hospital and municipality) |  | 1. continuity of care 2. Teams 3. comprehensive geriatric assessment 4. shared decision making 5. discharge planning | 1. Use of information and communication technology | 1. Local consensus processes 2. continuous quality improvement | 1. direct care delivery  2. organizational design and governance - by representation at the PACT board that represents significant stakeholders: Patients, primary and secondary care, research, and health technology | Use of health services | Design of interventions |  |
|  | Person-centred care |  |  |  |  |  |  |  |  | Outcomes of care | Selection of beneficiaries and allocation of intervention to practices |  |
|  | Behavioural change techniques |  |  |  |  |  |  |  |  |  | Operationalization of measures |  |
| Does a social prescribing 'holistic' link-worker for older people with complex, multimorbidity improve well-being and frailty and reduce health and social care use and costs? A 12-month before-and-after evaluation | Person-centred approach | aged 50 years or over, two or more long-term conditions | Micro - clinical | community based facilities | 1. Self-management | 1. Case management |  | 1. Educational meetings |  | Process of care Use of health services | Design of interventions |  |
|  |  |  |  |  |  |  |  |  |  | Outcomes of care | Selection of beneficiaries and allocation of intervention to practices |  |
|  |  |  |  |  |  |  |  |  | 1. direct care delivery |  | Size and timing effects |  |
| †Management of multimorbidity using a patient-centred care model: a pragmatic cluster-randomised trial of the 3D approach | Chronic care model | aged 18 years or older, at least three types of chronic condition (type of condition specified), grouped into ten types of condition with similar management considerations | Meso - professional | general practices |  | 1. Continuity of care 2. Teams | 1. The use of information and communication technology | 1. Educational meetings 2. Monitoring the performance of the delivery of healthcare | 1. direct care delivery 2. organizational design and governance – a PPI group was set up to align the intervention with he perceived needs of people with multimorbidity | Process of care | Design of interventions |  |
|  | Patient­-centred care model international guidelines |  | Micro - clinical |  |  |  |  |  |  | Use of health services | Selection of beneficiaries and allocation of intervention to practices |  |
|  |  |  |  |  |  |  |  |  |  | Experience of care and satisfaction | Operationalization of measures |  |
|  |  |  |  |  |  |  |  |  |  | Outcomes of care | Size and timing of effects |  |
|  |  |  |  |  |  |  |  |  |  |  |  |  |
| †Can implementation failure or intervention failure explain the result of the 3D multimorbidity trial in general practice: Mixed methods process evaluation |  |  |  |  |  |  |  |  |  | n.ap. | Design of interventions |  |
|  |  |  |  |  |  |  |  |  |  |  | Selection of beneficiaries and allocation of intervention practices |  |
|  |  |  |  |  |  |  |  |  |  |  | Size and timing of effects |  |
|  |  |  |  |  |  |  |  |  |  |  | Change management and implementation - feasibility |  |
|  |  |  |  |  |  |  |  |  |  |  |  |  |
| Is telephone health coaching a useful population health strategy for supporting older people with multimorbidity? An evaluation of reach, effectiveness and cost-effectiveness using a 'trial within a cohort' | Behavioural change theories and techniques | aged over 65, two or more self-reported long-term conditions, needing assistance with self-management (moderate activation) | Micro - clinical | via telephone from a central NHS facility | 1.self-management |  | 1. electronic assistive technologies | 1. educational meetings 2- educational outreach visits, or academic detailing 3- educational materials | 1- direct care delivery 2- organizational design and governance - Members of local groups were consulted about the health coaching model, recruitment and retention methods | Process of care | Selection of beneficiaries and allocation of intervention to practices |  |
|  |  |  |  |  |  |  |  |  |  | Use of health services | Operationalization of measures |  |
|  |  |  |  |  |  | 1- continuity of care |  |  |  | Outcomes of care | Size and timing of effects |  |
|  |  |  |  |  |  |  |  |  |  |  | Change management and implementation - feasibility |  |
| A self-management support program for older Australians with multiple chronic conditions: A randomised controlled trial | Stanford Chronic Disease Self-Management Model | aged 60 years or more, two chronic conditions, and rated health differently from “very good” or “excellent” | Micro - clinical | clinician led CDSMS program | 1.self-management |  |  | 1. Educational outreach visits, or academic detailing | 1. direct care delivery | Process of care Use of health services | Design of interventions |  |
|  | Behavioural change theories |  |  |  |  |  |  |  |  | Outcomes of care | Selection of beneficiaries and allocation of intervention to practices |  |
|  |  |  |  |  |  |  |  |  |  |  | Operationalization of measures |  |
|  |  |  |  |  |  |  |  |  |  |  | Size and timing of effects |  |
|  |  |  |  |  |  |  |  |  |  |  | Change management and implementation - feasibility |  |
| †Effectiveness and cost-effectiveness of a nurse-delivered intervention to improve adherence to treatment for HIV: a pragmatic, multicentre, open-label, randomised clinical trial | Behavioural change theories | HIV, treatment experienced at risk of viral rebound or treatment-naive | Micro – clinical | HIV clinics at academic and non-academic hospitals | 1.self-management |  | 1. smart home technologies | 1. educational meetings | 1. direct care delivery | Process of care Use of health services | Design of interventions | x |
|  |  |  |  |  |  |  |  |  |  | Outcomes of care | Change management and implementation – acceptability, feasibility |  |
| †Cost-effectiveness and Cost-utility of the Adherence Improving Self-Management Strategy in Human Immunodeficiency Virus Care: A Trial-based Economic Evaluation |  |  |  |  |  |  |  |  |  | Process of care Use of health services | Operationalization of measures |  |
|  |  |  |  |  |  |  |  |  |  | Outcomes of care | Size and timing of effects |  |
|  |  |  |  |  |  |  |  |  |  |  | Change management and implementation – feasibility |  |
|  |  |  |  |  |  |  |  |  |  |  |  |  |
| The systematic development of a complex intervention: HealthMap, an online self-management support program for people with HIV | Chronic Care Model | HIV, aged 30 or over, excluding those diagnosed with cardiovascular disease | Micro - clinical | online | 1.self-management | 1. continuity of care | 1. the use of information and communication technology |  | 1. direct care delivery 2. organizational design and governance - needs assessment via concept mapping workshops, online surveys and interviews with PLHIV and care providers | n.ap. | Design of interventions |  |
|  | Behavioural change theories Chronic Disease Self-Management models |  |  |  |  |  |  |  |  |  | Selection of beneficiaries and allocation of intervention to practices |  |
|  | Precede- proceed Model |  |  |  |  |  |  |  |  |  | Change management and implementation – acceptability, feasibility, transferability |  |
|  |  |  |  |  |  |  |  |  |  |  |  |  |
| Qualitative Evaluation of the Implementation of an Integrated Care Delivery Model for Chronic Patients with Multi-Morbidity in the Basque Country | Chronic Care Model | multiple diseases and/or on multiple medications, at the top of the risk stratification | Micro - clinical | integrated organization - regional hospital and primary care centres | 1. role expansion or task shifting | 1. role expansion or task shifting |  |  | n.a. | n.ap. | Design of interventions | x |
|  | Triple Aim |  |  |  |  |  |  |  |  |  | Change management and implementation – acceptancy and feasibility |  |
|  |  |  |  |  |  |  |  |  |  |  |  |  |
| Feasibility and Preliminary Outcomes of a Web and Smartphone-Based Medication Self-Management Platform for Chronically Ill Patients | n.a. | well controlled HIV patients (undetectable viral loads and with follow-up coordinated between primary and secondary care) | Micro - clinical | online | 1. self-management |  | 1. the use of information and communication technology |  | 1. direct care delivery 2. organizational design and governance - along different phases as platform requirements analysis, and validation process | Process of care Use of health services | Selection of beneficiaries and allocation of intervention to practices | x |
|  |  |  |  |  |  |  |  |  |  | Experience of care and satisfaction | Operationalization of measures |  |
|  |  |  |  |  |  |  |  |  |  | Outcomes of care | Size and timing of results |  |
|  |  |  |  |  |  |  |  |  |  |  | Change management and implementation – acceptancy and feasibility |  |
|  |  |  |  |  |  |  |  |  |  |  |  |  |
| mHealth Tools for the Self-Management of Patients with Multimorbidity in Primary Care Settings: Pilot Study to Explore User Experience | Individual and Family Self-Management Theory | multiple chronic conditions and social complexity | Micro - clinical | primary care setting | 1. self-management |  | 1. The use of information and communication technology | 1. educational meetings | 1. direct care delivery  2. organizational design and governance - the ePRO tool was codeveloped with patients and providers using a user-centred design approach - interviews and working groups | n.ap. | Selection of beneficiaries and allocation of intervention to practices | x |
|  |  |  |  |  |  |  |  |  |  |  | Operationalization of measures |  |
|  |  |  |  |  |  |  |  |  |  |  | Size and timing of effects |  |
|  |  |  |  |  |  |  |  |  |  |  | Change management and implementation – acceptancy and feasibility |  |
|  |  |  |  |  |  |  |  |  |  |  |  |  |
| †Online self-management for gay men living with HIV: a pilot study | Behavioural change theories | HIV diagnosis, male sex, age over 18 years | Micro - clinical | online | 1. self-management |  | 1. The use of information and communication technology | 1. educational meetings 2. educational materials | 1. direct care delivery  2. organizational design and governance - needs assessment combining qualitative and quantitative methodologies | Process of care Experience of care and satisfaction | Design of interventions | x |
|  | Chronic Disease Self-Management framework |  |  |  |  |  |  |  |  | Outcomes of care | Operationalization of measures |  |
|  | Precede- proceed Model |  |  |  |  |  |  |  |  |  | Change management and implementation – acceptancy and feasibility |  |
|  |  |  |  |  |  |  |  |  |  |  |  |  |
| †The Positive Outlook Study: A Randomised Controlled Trial Evaluating Online Self-Management for HIV Positive Gay Men |  |  |  |  |  |  |  |  |  | Process of care Experience of care and satisfaction | Design of interventions |  |
|  |  |  |  |  |  |  |  |  |  | Outcomes of care | Selection of beneficiaries and allocation of intervention to practices |  |
|  |  |  |  |  |  |  |  |  |  |  | Operationalization of measures |  |
|  |  |  |  |  |  |  |  |  |  |  | Size and timing of effects |  |
|  |  |  |  |  |  |  |  |  |  |  | Change management and implementation – acceptancy and feasibility |  |

Legend of table: †studies associated to the same intervention; n.a. not available; n.ap. not applicable.
